# Editorial: Advances in beneficial and pathogenic plant-microbe interactions in cereal crops

**DOI:** 10.3389/fmicb.2025.1663889

**Published:** 2025-08-06

**Authors:** Febri Doni, Jian Chen, Kumudini Belur Satyan

**Affiliations:** ^1^Department of Biology, Faculty of Mathematics and Natural Sciences, Universitas Padjadjaran, Jatinangor, West Java, Indonesia; ^2^Department of Global Development, Cornell University, New York, NY, United States; ^3^International Genome Center, Jiangsu University, Zhenjiang, China; ^4^Department of Biotechnology and Genetics, School of Sciences, JAIN (Deemed-to-be University), Bengaluru, Karnataka, India

**Keywords:** plant-microbe interactions, pathogenic interactions, symbiosis, plant diseases, omics technologies, cereal crops, sustainable crop production

## Introduction

Plant-microbe interactions play a pivotal role in shaping the health, productivity, and resilience of cereal crops such as wheat and rice (Akbari et al., 2024). These complex relationships are central to agricultural ecosystem functioning and hold great potential for sustainable crop production (Harman et al., 2021). This Research Topic highlights recent advances in our understanding of both beneficial and pathogenic plant-microbe interactions, focusing on cereal crops including wheat, maize, rice, barley, oats, rye, and sorghum.

## Developing sustainable microbial inoculants for cereal crops

Arbuscular mycorrhizal fungi (AMF) have long been recognized for their symbiotic associations with plant roots (Ibáñez et al., 2025). Boyno et al. provide a comprehensive review of the roles of AMF in improving nutrient uptake, promoting stress resilience, and activating systemic resistance in host plants. They synthesize molecular and field data showing AMF's potential to enhance both abiotic and biotic stress tolerance. However, their effectiveness is still limited in field settings due to factors such as host specificity, soil characteristics, and environmental variability.

Alattas et al. focus on *Pseudomonas* spp., a diverse bacterial genus known for its biocontrol capabilities. The review details mechanisms by which *Pseudomonas* suppresses plant pathogens, including antimicrobial production, volatile compound emission, and immune response activation. Nonetheless, field application remains inconsistent due to environmental fluctuations and microbial competition in the rhizosphere.

Addressing abiotic stress, particularly drought, Niaz et al. explore a fungal consortium of *Aspergillus oryzae* and *A. fumigatus* adapted to drought conditions. Their study shows improved drought tolerance in maize through enhanced antioxidant activity, phytohormonal regulation, and upregulation of stress-responsive genes.

Phosphorus management is another critical issue. de Oliveira-Paiva et al. evaluate phosphate-solubilizing *Bacillus* strains (*B. megaterium* and *B. subtilis*) in multi-season field trials in Brazil. Results demonstrated significant improvements in phosphorus uptake and yield, leading to the registration of the first *Bacillus*-based maize inoculant in the country. Additionally, Huang et al. assess a low-temperature straw-degrading microbial consortium containing *Pseudogymnoascus* sp. in winter wheat fields. Their findings show enhanced soil fertility, wheat yield, and changes in microbial community structure, especially in sandy soils.

## Multi-omics and machine learning to elucidate plant-microbe interactions

Omics technologies have become indispensable for decoding plant-microbe interactions (Doni et al., 2022). Ejaz et al. present a comparative genomics study using long-read sequencing to analyze *Xanthomonas oryzae* pv. *oryzae* isolates from Pakistani Basmati rice. The study finds high similarity with Indian and Thai strains, contributing to effector-based surveillance strategies in breeding programs.

Mekonen et al. analyze 25 *Colletotrichum sublineola* isolates from Ethiopian sorghum fields, revealing virulence variability and identifying resistant genotypes like IS_18760 and Bonsa. These insights are crucial for developing anthracnose-resistant varieties in East Africa. Yang et al. examine root and rhizosphere microbiomes in healthy and *Rhizoctonia solani*-infected potato plants across Chinese agroecological zones. Healthy plants showed more diverse, cooperative microbial networks with disease-suppressive potential.

Castano-Duque et al. integrate satellite imagery, weather, and soil data to predict aflatoxin outbreaks in Texas maize using neural network-based models. Their best model achieved 73% accuracy and identified high-risk zones, reinforcing the value of predictive frameworks that incorporate phenology and hydrology for site-specific risk mitigation.

## Conclusion

This Research Topic offers diverse studies that deepen our understanding of plant- microbe interactions in cereal cropping systems. The featured contributions—from microbial inoculants to omics technologies and predictive modeling—underscore the importance of microbial approaches in fostering resilience, productivity, and sustainability in cereal agriculture.

## References

[B1] Akbari S. I. Prismantoro D. Permadi N. Rossiana N. Miranti M. Mispan M. S.. (2024). Bioprospecting the roles of *Trichoderma* in alleviating plants' drought tolerance: principles, mechanisms of action, and prospects. Microbiol. Res. 283:127665. 10.1016/j.micres.2024.127665 38452552

[B2] Doni F. Miranti M. Mispan M. S. Mohamed Z. Uphoff N. (2022). Multi-omics approaches for deciphering the microbial modulation of plants' genetic potentials: what's known and what's next?. Rhizosphere 24:100613. 10.1016/j.rhisph.2022.100613

[B3] Harman G. Khadka R. Doni F. Uphoff N. (2021). Benefits to plant health and productivity from enhancing plant microbial symbionts. Front. Plant Sci. 11:610065. 10.3389/fpls.2020.610065 33912198 PMC8072474

[B4] Ibáñez I. McPherson M. R. Upchurch R. A. Zak D. R. (2025). Mycorrhizal fungi influence on mature tree growth: stronger in high-nitrogen soils for an emf-associated tree and in low-nitrogen soils for two amf-associated trees. Plant Environ. Interac. 6:e70055. 10.1002/pei3.70055 40342515 PMC12059558

